# Developmental delay and its predictors among children under five years of age with uncomplicated severe acute malnutrition: a cross-sectional study in rural Pakistan

**DOI:** 10.1186/s12889-021-11445-w

**Published:** 2021-07-15

**Authors:** Javeria Saleem, Rubeena Zakar, Gul Mehar Javaid Bukhari, Aneela Fatima, Florian Fischer

**Affiliations:** 1 Department of Public Health, Institute of Social and Cultural Studies, University of the Punjab, Lahore, Pakistan; 2 Department of Community Medicine, Federal Medical and Dental College, Islamabad, Pakistan; 3 Pakistan Council of Scientific and Industrial Research, Islamabad, Pakistan; 4 Institute of Public Health, Charité – Universitätsmedizin Berlin, Berlin, Germany; 5 Institute of Gerontological Health Services and Nursing Research, Ravensburg-Weingarten University of Applied Sciences, Weingarten, Germany

**Keywords:** Developmental delay, Severe acute malnutrition, Wasting, Stunting, Children

## Abstract

**Background:**

In developing countries, malnutrition in children and developmental delays are two major challenges for public health. To achieve the vision of the Sustainable Development Goals from the broader perspective of child health, early identification of developmental delays and timely intervention are crucial. The aim of this study is to assess the prevalence of suspected developmental delay and their predictors in children under the age of 5 years with uncomplicated severe acute malnutrition in rural areas of Pakistan.

**Methods:**

A multicentre cross-sectional study was conducted among 185 children with uncomplicated severe acute malnutrition. We screened children aged 6–59 months for their nutritional status and clinical complications. Children fulfilling the inclusion criteria underwent the Denver Development Screening Tool II (DDST-II). The children’s global developmental profile was calculated according to the established protocols of DDST-II, which are based on four important domains of development: personal and social behaviour, language, gross motor adaptive skills and fine motor adaptive skills. A pretested questionnaire was used to collect data on socio-demographic and nutritional factors for assessing predictors of developmental delay, which were analysed using a multivariate logistic regression model.

**Results:**

Out of 177 children with severe acute malnutrition, 69 (38.9%) had normal global development and 108 (61.1%) had delayed global development. Significant associations were found between global developmental delay and younger children (6–24 months vs. 25–59 months; AOR = 4.53, 95% CI: 1.56–13.10, *p* < 0.01), children who were not exclusively breastfed (AOR = 3.07, 95% CI: 1.24–7.56, *p* = 0.01), and a history of contact with a tuberculosis smear-positive adult (AOR = 2.67, 95% CI: 1.30–5.49, *p* < 0.01).

**Conclusion:**

About two thirds of the study participants showed delayed or unstable global development. Thus, according to DDST-II-established protocols, there is a high prevalence of suspected developmental delay among children under the age of five years with uncomplicated severe acute malnutrition in rural areas of Pakistan. Children in their first 2 years of life were at particularly high risk due to insufficient breastfeeding. This emphasizes the need to provide adequate infrastructure and information to parents for the prevention of developmental delay in remote areas.

## Background

Developmental delay indicates extensive deficits and lack of developmental skills that would be appropriate for children at a particular age. It may be exhibited in different domains, including motor adaptive skills, language or personal and social behaviour [1, 2]. Grantham et al. estimated that, globally, just over 200 million children under five are unable to attain their developmental potential due to poverty, malnutrition, inappropriate childcare and/or child abuse. Most of these were from South Asia and sub-Saharan Africa [3].

Severe acute malnutrition (SAM), also known as severe wasting, represents the most critical form of undernutrition. This is further classified into complicated or uncomplicated SAM, based on the presence or absence of medical complications [4]. Children with medically complicated SAM will mostly be treated as inpatients at a medical facility, but children with uncomplicated SAM are advised to be nursed at home with high-calorie, micronutrient-enriched food [5]. SAM is responsible for a large proportion of the global disease burden among children and has a high lethality (10–30%). Those children who survive have compromised physical and cognitive development, which leads to reduced productivity in adulthood [6–8].

Early childhood, referring to the first 5 years of life, is the fastest and most sensitive period of child growth and brain development. This period is easily influenced by poverty and biological and psychosocial risk factors [1, 9]. Prior studies have shown strong associations between nutritional status and developmental outcomes in developing countries [10–12]. Malnutrition constrains rapid brain development by adversely affecting its structural and functional capacity, resulting in developmental deficits among the children in all domains [3, 13]. An unhealthy external environment also has a negative impact on children’s ability to learn social and developmental skills [11]. Therefore, several social, biological and psychological factors can contribute to developmental delay in children [1].

Monitoring the health and nutritional status of children under five years of age is important to ensure that they achieve their developmental potential, including both physical and psychological well-being [14]. It is also helpful in achieving the Sustainable Development Goals (SDGs), particularly SDG 3 and SDG 4, from the broader perspective of child health beyond mere survival. Globally, an estimated 19 million children suffer from SAM. Of these, around 1.4 million are from Pakistan [6]. Like other developing countries, Pakistan is struggling with the public health issue of child malnutrition [15]. According to the Pakistan Demographic Survey (2017–18), 37.6% of children aged under five were stunted, with 17% severely stunted. Furthermore, 7.1% were found to be wasted, with 2% severely wasted; and 23% were underweight [16]. According to the Multiple Indicator Cluster Survey (MICS), Dera Ghazi Khan had the highest prevalence of stunting (41%) in Punjab [17].

Although sufficient data is available on the nutritional profile of children aged under five in Pakistan, there is still a scarcity of data on the developmental potential of malnourished children. Moreover, the factors influencing child development delays and the associations between these factors in children with severe acute malnutrition have seldom been examined. Therefore, this study aims to assess the prevalence of developmental delays and their predictors in children under the age of five years with uncomplicated severe acute malnutrition in rural Pakistan.

## Methods

### Study design

A multicentre cross-sectional study was conducted during a one-year period, from November 2016 to November 2017.

### Study area

The study was conducted at the Outpatient Therapeutic Programme (OTP) Centres in the District of Dera Ghazi (DG) Khan in Southern Punjab, Pakistan. DG Khan has the worst indicators of malnutrition (stunting and wasting) in Punjab. According to MICS 2018, 41% of children under five were stunted and 7.5% were wasted in DG Khan [17]. This socio-economically underprivileged district has the highest prevalence of illiteracy (65% of women and 38.5% of men aged 15–49 were illiterate [17]). Overcrowding is a problem, and the area is frequently affected by floods [17, 18].

Out of a total of 16 OTPs in DG Khan region, four centres were selected on the recommendation of the District Health Office. The reason for recommending these four centres was that they were functioning more actively than the others because appropriate numbers of staff and sufficient therapeutic food were available there. These OTP centres were being used for screening the nutritional status of children and the timely recognition and referral of complicated cases to inpatient facilities. Children with acute and moderate malnutrition without complications were treated at these centres with Ready to Use Therapeutic Food (RUTF) as a community-based treatment. These OTP centres were also used to provide nutritional supplements to pregnant and lactating mothers.

### Study population

Overall, 185 children, aged 6–59 months, were selected to participate in the study on condition of receiving their parents’ written informed consent. At enrolment, these children had SAM without complications according to World Health Organization (WHO) criteria (mid-upper arm circumference [MUAC] < 115 mm and a weight-for-height [Z score] < − 3), were clinically well, alert and had a good appetite. Children with SAM and complications were not included in the study on baseline assessment. Complications, as defined by the WHO, were hypoglycaemia, hypothermia (axillary temperature < 35 °C), hyperpyrexia (axillary temperature > 39 °C), anorexia, severe dehydration, grade three pitting oedema, severe anaemia and acute lower respiratory tract infection [5]. Children who were clinically ill due to any of these symptoms or having poor appetite were not included in the study. Instead, they were referred to an inpatient facility for further evaluation and treatment. Children aged over 59 months or below 6 months were also not included. Parents’ refusal to participate in the study and a failure to assess a child’s developmental status were also exclusion criteria.

### Sample size

**Fig. 1:**
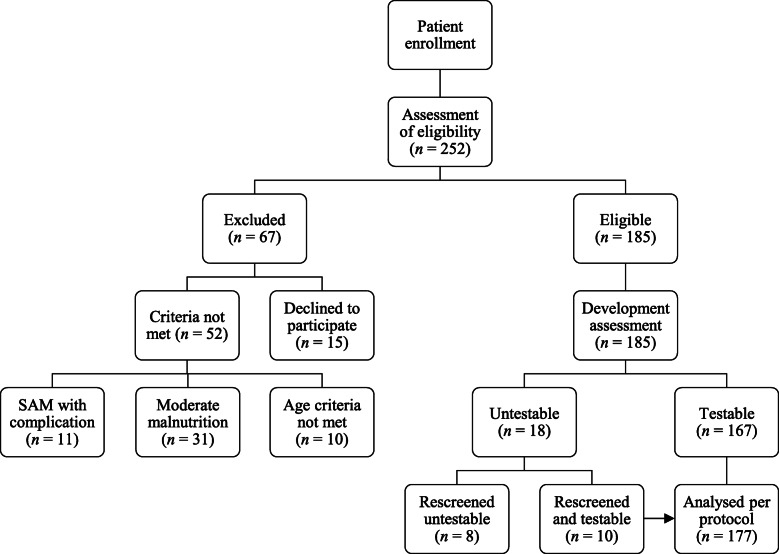
Flowchart of sample selection and developmental screening

For the sample size calculation, WHO Sample Size 2.0 and the following formula were used:

$$ n=\frac{Z^2\bullet P\bullet \left(1-P\right)}{d^2} $$

with a level of significance of 95% (Z = 1.96), an expected proportion of wasting (P) of 11% [17], and an expected error (d) of 5%. This leads to a sample size of 151, which was increased to a total of 185 to enhance the study’s precision, strength and accuracy. The eligibility to participate in the study was assessed among 252 children, of whom 67 were excluded, because either they did not meet the inclusion criteria (*n* = 52), or their parents refused to participate (*n* = 15) (Fig. 1).

### Data collection

Study data were collected in three steps. Firstly, an enrolment form comprising anthropometry measurements and medical records (history and physical examination) was filled in by a qualified medical practitioner at the relevant centre. Secondly, a structured and pretested questionnaire was administered by the same medical practitioner to access data on socio-demographic characteristics (family, income, education etc.) and patterns of food consumption. Thirdly, the Denver Development Screening Tool (DDST-II) was used for the development assessment of children by the paediatrician, who was especially deputed for this purpose.

The gestational age of mothers was taken from antenatal records in cases of hospital delivery, or based on maternal recall if the birth took place at home. The corrected age of children ≤24 months who were born prematurely (before 37 weeks of gestation) was used. Corrected age was calculated by deducting the number of weeks of missed gestation from the present age.

Medical history was obtained for symptoms of acute diarrhoea, high-grade fever, lethargy, coughing, shortness of breath, seizures, neurological deficit and anorexia. All of these symptoms were indications of a child being clinically ill. In physical examination, severe dehydration, palmar pallor (as an indication of anaemia), and severe pitting oedema were assessed in the children [4, 5]. Their vital signs were assessed for hypothermia/hyperpyrexia and respiratory rate. For the assessment of hypoglycaemia, a heel-prick was done and children with glucose concentrations of < 3 mmol/L based on the Dextrostix reagent strip by Roche were deemed to be hypoglycaemic. An appetite test was conducted by giving the child a small amount of RUTF (Plumpy Nut by Nutriset) to eat. A child who did not consume a minimum of one third of a packet (three teaspoons, 30 g) of RUTF after three tries was labelled as having poor appetite [4–6]. These children were not included in the study.

### Measurements

#### Anthropometric measurements

Anthropometric measurements were conducted by the medical staff at the participating centres, who were specifically trained in child nutritional assessment by the master trainers from the nutrition programme of the Provincial Health Department. Assessments were made twice: first by a trained Lady Health Worker (LHW) and then by the Medical Officer of the centre to avoid potential bias. In cases where the two measurements differed, further assessments were conducted until a precise value was obtained. The repeated value was then documented. The mid upper arm circumference (MUAC) was assessed to the nearest 0.1 cm with colour-labelled MUAC tape at the midpoint between the olecranon and the acromion process.

Child weight was measured using UNISCALE (SECA 878), to the nearest 10 g, undressed or in a light dress. If children or infants were not able to stand by themselves, UNISCALE was utilized to determine only the mothers’ weight. Afterwards, the infant/child was handed to the mother while she was standing on the scale and the collective infant and mother weight was assessed. The calculation of infant/child weight was determined as the difference between these two readings. UNISCALE was calibrated for standard weight and adjusted to zero prior to all measurements.

The recumbent length of children who were less than or equal to 87 cm in height was assessed to the nearest 0.1 cm with the help of a length-measuring board with a fixed headrest and a mobile foot piece (“SECA GmbH & Co. KG, Hamburg, Germany”), laid on a smooth surface in supine position. Children taller than 87 cm in height were assessed using Stadiometer (SECA 217) in a standing position after removing shoes and with heels together on a horizontal flat plate attached to the base of the measuring board [18]. Standard protocols for child growth were applied for calculating the weight-for-height Z-score using WHO ANTHRO version 3.2.2.

#### Developmental screening

Study participants underwent the DDST-II [19] in order to assess their development profile. This tool measures the proficiency of children under the age of six years to do a range of different tasks and makes comparisons with a standardized population of same-age children. It measures the four parameters of development: personal-social behaviour development, fine motor development, language, and gross motor development. This tool was applied by a physician trained in conducting DDST-II for child development assessment. The average screening time for DDST-II was about 30 min. If the child was uncooperative and untestable at the first screening for global developmental status, a follow-up rescreen visit was conducted after two days. If the child was still untestable, the screening was repeated after a further two days. The tool was translated into the child’s native language in order to eliminate communication barriers. The flowchart of developmental screening is presented in Fig. 1.

Following a standardized algorithm, each child’s development was categorized as normal, if the child performs the items on the left of the age line completely. There is a further category entitled “caution”, which is an intermediate classification, for children who are unsuccessful to an item on which the age line falls on or between the 75th and 90th percentiles. Finally, children are categorized as delayed, if they do not pass an item that 90% of children in the standardization passed at an earlier age and item on which age line falls completely to the left of the age line [19].

These category measurements were then applied to grade global developmental status as “normal” (no category delayed and no more than one category classified as caution), “suspect” (≥2 cautions or ≥ 1 delay), or “untestable” (based on a specific pattern of refusals) [19].

### Statistical analyses

Data was analysed using SPSS version 23. Simple logistic regression was applied to assess the bivariate relationship through unadjusted Odds Ratios (OR) between the dependent and independent variables. The *p*-value was set at 0.20 in order to include all significant variables as confounding variables in the multivariate analysis [20]. Hence, a multivariate logistic regression was run to measure the dynamics among the potential predictors for delayed development among the children and to present the results in terms of Adjusted Odds Ratios (AOR) with 95% confidence intervals (95% CI) and level of significance (*p* < 0.05).

**Fig. 2:**
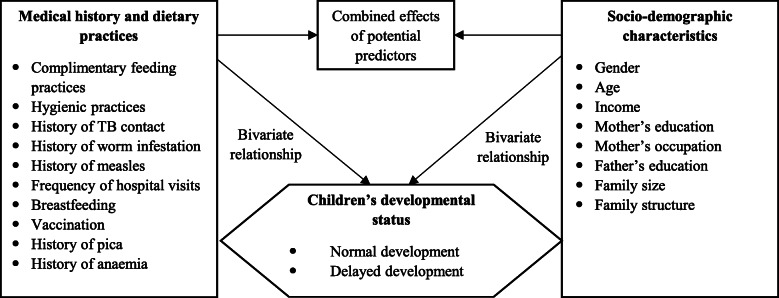
Relationships between variables affecting developmental delay

The outcome variable was the children’s developmental status as a binary variable (delayed development vs. normal development). Socio-demographic characteristics, medical history and dietary practices were included as independent variables, because they can theoretically affect developmental delay (Fig. 2).

## Results

### Developmental status

Out of the total 185 children, eight (4.3%) were untestable for developmental status even after rescreening. Therefore, the analysis is based on 177 children. The results for the prevalence of suspected global development delays based on developmental milestones, in all domains and separated for each domain, are presented in Table 1. According to this, 61.1% (*n* = 108) showed a suspected delay at the global level. The delay was most pronounced in personal and social behaviour (62.1%, *n* = 110), whereas for language 70.6% (*n* = 125) showed normal development.

**Table 1:** Prevalence of suspected global developmental delay (*n* = 177)

	***Categories***	***n (%)***
Global development	Normal Suspected delay	69 (38.9) 108 (61.1)
Development in domains		
Personal and social behaviour	Normal	67 (37.9)
	Delay	81 (45.8)
	Caution	29 (16.3)
Fine motor	Normal	113 (63.8)
	Delay	43 (24.3)
	Caution	21 (11.9)
Language	Normal	125 (70.6)
	Delay	23 (13.0)
	Caution	29 (16.4)
Gross motor	Normal	115 (65.0)
	Delay	28 (15.8)
	Caution	34 (19.2)

### Socio-demographic characteristics and developmental status

Comparisons of socio-demographic characteristics between children with normal development and suspected developmental delay are presented in Table 2. The comparison revealed that child’s age (*p* < 0.01), monthly income (*p* = 0.04), mother’s education (*p* = 0.01), father’s education (*p* = 0.04) and family size were (*p* = 0.01) significantly associated with suspected developmental delay. All other socio-demographic factors showed no significant association with developmental status.

**Table 2:** Socio-demographic characteristics and developmental status (*n* = 177)

***Socio-demographic characteristics***	***Categories***	***n***	***Normal development*** ***n (%)***	***Delayed development*** ***n (%)***	***OR (95% CI)***	***p-value***
Sex	Male	78	27 (34.6)	51 (65.4)	1.39 (0.75–2.57)	0.29
	Female	99	42 (42.4)	57 (57.6)	Reference	
Age (in months)	6–24	151	50 (33.1)	101 (66.9)	5.48 (2.16–13.9)	< 0.01
	25–59	26	19 (73.1)	7 (26.9)	Reference	
Monthly income (in PKR)	< 15,000	121	41 (33.9)	80 (66.1)	1.95 (1.02–3.72)	0.04
	≥ 15,000	56	28 (50.0)	28 (50.0)	Reference	
Mother’s education	Illiterate	128	43 (33.6)	85 (66.4)	2.23 (1.14–4.36)	0.01
	≥ Primary	49	26 (53.1)	23 (46.9)	Reference	
Mother’s occupation	Working	19	10 (52.6)	9 (47.4)	Reference	0.20
	Housewife	158	59 (37.3)	99 (62.7)	1.86 (0.72–4.85)	
Father’s education	Illiterate	109	36 (33.0)	73 (67.0)	1.91 (1.02–3.55)	0.04
	≥ Primary	68	33 (48.5)	35 (51.5)	Reference	
Family size	< 8	40	22 (55.0)	18 (45.0)	Reference	0.01
	> 8	137	47 (34.3)	90 (65.7)	2.34 (1.14–4.79)	
Family structure	Joint	72	30 (41.7)	42 (58.3)	Reference	0.54
	Nuclear	105	39 (37.1)	66 (62.9)	1.21 (0.65–2.32)	

### Medical history, dietary practices and developmental status

A significant association with delayed development was found for the frequency of hospital visits (*p* = 0.03), which are visits to hospital in the past 6 months due to diarrhoea, respiratory infections or because of any other illness (which was confirmed from their medical records to avoid any recall bias); history of tuberculosis (TB) contact (*p* = 0.01) (defined as a child with close contact to a TB smear-positive adult patient at home or in the near surroundings); and breastfeeding practices (*p* = 0.01). Furthermore, a history of worm infestation (*p* = 0.14) was included in the multivariate analysis as the level of significance was set at *p* < 0.20 for this analysis. All other variables, such as complementary feeding practices (complementary food was probed in detail from mothers according to WHO recommendations by showing them different utensils and food charts to assess quantity, variety and frequency of feeding), hygiene practices, vaccination, and history of measles, were shown to not be related to delayed development among children (Table 3).

**Table 3:** Medical history, dietary practices and developmental status (*n* = 177)

***Medical history and dietary practices***	***Categories***	***n***	***Normal development*** ***n (%)***	***Delayed development*** ***n (%)***	***OR (95% CI)***	***p-value***
Complementary feeding practices	Poor	136	18 (43.9)	23 (56.1)	1.30 (0.64–2.65)	0.46
	Good	41	51 (37.5)	85 (62.5)	Reference	
Hygienic practices	Poor	157	8 (40.0)	12 (60.0)	1.05 (0.41–2.71)	0.92
	Good	20	61 (38.9)	96 (61.1)	Reference	
History of TB contact	Yes	77	22 (28.6)	55 (71.4)	2.22 (1.18–4.17)	0.01
	No	100	47 (47.0)	53 (53.0)	Reference	
History of worm infestation	Yes	41	12 (29.3)	29 (70.7)	1.74 (0.82–3.71)	0.14
	No	136	57 (41.9)	79 (58.1)	Reference	
History of measles	Yes	26	9 (34.6)	17 (65.4)	1.24 (0.52–2.98)	0.62
	No	151	60 (39.7)	91 (60.3)	Reference	
Frequency of hospital visits	1–7	95	44 (46.3)	51 (53.7)	Reference	0.03
	8–15	82	25 (30.5)	57 (69.5)	1.96 (1.05–3.65)	
Breastfeeding	Mixed	143	49 (34.3)	94 (65.7)	2.74 (1.27–5.89)	0.01
	Exclusive	34	20 (58.8)	14 (41.2)	Reference	
Vaccination	Incomplete	45	14 (31.1)	31 (68.9)	1.58 (0.77–3.24)	0.21
	Complete	132	55 (41.7)	77 (58.3)	Reference	
History of pica	Yes	58	22 (37.9)	36 (62.1)	1.06 (0.56–2.03)	0.84
	No	119	47 (39.5)	72 (60.5)	Reference	
History of anaemia	Yes	76	31 (40.8)	45 (59.2)	0.87 (0.70–1.61)	0.66
	No	101	38 (37.6)	63 (62.4)	Reference	

### Multivariate logistic regression

All variables that were found to be significant (*p* ≤ 0.20) in the simple logistic regression models (Tables 2 and 3) were included in the multivariate logistic regression analysis. As multicollinearity was found between mother’s and father’s education, we only kept mother’s education in the analysis to account for the fact that mother’s education has been found to be a significant predictor of child development in previous studies [21].

In the multivariate logistic regression model, only a younger age (6–24 months vs. 25–59 months) of the child (AOR = 4.53, 95% CI: 1.56–13.10, *p* < 0.01), history of TB contact (AOR = 2.67, 95% CI: 1.30–5.49, *p* < 0.01), and mixed breastfeeding practices (AOR = 3.07, 95% CI: 1.24–7.56, *p* = 0.01) remained significantly associated with developmental delays (Table 4).

**Table 4:** Multivariate logistic regression for predictors of developmental delay (*n* = 177)

***Independent variables***	***Categories***	***AOR (95% CI)***	***p-value***
Age (in months)	6–24	4.53 (1.56–13.10)	< 0.01
	25–59	Reference	
Monthly income (in PKR)	< 15,000	1.43 (0.64–3.17)	0.37
	≥15,000	Reference	
Mother’s education	Illiterate	1.65 (0.75–3.62)	0.21
	≥Primary	Reference	
Mother’s occupation	Working	Reference	0.22
	Housewife	0.50 (0.16–1.52)	
Family size	< 8	Reference	0.14
	> 8	1.87 (0.80–4.40)	
History of TB contact	Yes	2.67 (1.30–5.49)	< 0.01
	No	Reference	
History of worm infestation	Yes	2.02 (0.85–4.81)	0.11
	No	Reference	
Frequency of hospital visits	1–7	Reference	0.10
	8–15	1.87 (0.88–3.97)	
Breastfeeding	Mixed	3.07 (1.24–7.56)	0.01
	Exclusive	Reference	

## Discussion

This study revealed a significant prevalence of suspected developmental delays (61.1%) among children aged under five with severe acute malnutrition. Malnutrition in children is one of the main reasons for developmental delay, which leads to a lifelong adverse impact on health [10, 18]. Undernutrition affects a child’s behaviour and temperament. It is also one of the reasons for low economic productivity in adulthood [18, 22]. Due to being lethargic and apathetic, malnourished children have problems in understanding information and they are less interested in their surroundings than well-nourished children. This results in delayed social interaction skills. Personal and social development during the initial years of life is the main factor responsible for desirable mental health and good performance in adulthood [18, 22]. Therefore, the recognition of personal, social and behavioural problems in young children is very important [23]. Within this study, we found that, in the domain of personal and social behaviour, almost half of the children (45.8%) had delayed development, with an additional 16.3% being in the caution zone. This result is in line with prevous literature and can, therefore, be generalized to other study settings [18, 22].

Children in our study were also found to be affected in language and motor adaptive skills. Previous research has explained that malnourished children also suffer from micronutrient deficiencies, such as calcium and vitamin D, which are important for skeletal muscle function. For that reason, a deficiency in these micronutrients could affect motor skills [6]. Nutritional insufficiencies at an acute stage may damage the cognitive profile and entire auditory system in children, resulting in verbal and written language problems. Previous studies have also reported a significant relationship between being underweight and delays in the development of motor and language skills among children [24, 25].

Child development proceeds through a gradual, multifaceted interaction between parental and caregivers’ education level, living and working conditions, social circumstances, availability of health facilities, and the physical environment. An unconstructive social or external environment during the early years of life is usually linked to compromised development. Every region or community has its own circumstances. For that reason, locally targeted research and interventions are needed to move forward [23]. In the present study, mother’s education, family’s monthly income, frequent hospital visits due to repeated illness, and family size were significantly linked to developmental delays in the bivariate analysis. This has also been shown in previous studies [1, 3, 10, 23].

Comparable research in Indonesian slum areas and in the United States have revealed that low maternal education and low family income are strongly associated with delayed development in young children [1, 26, 27]. This is due to the fact that maternal education has a direct effect on proper medical follow-up during the antenatal period, effective family planning, optimal nutrition and child healthcare [18]. The socioeconomic status of a family is one of the determinants of the nutritional status of its children. Children belonging to the lower socio-economic class were poorly nourished in comparison to those in the upper socio-economic class. This is due to food insufficiency and lack of food diversity at the household level. Children aged between one and 2 years were found to be significantly malnourished in comparison to older children [28].

In our study, exclusive breastfeeding was significantly associated with normal child development. A strong correlation between breastfeeding and progressive developmental outcomes in children has been proven previously [28, 29]. This can be explained by the nutritive value of breast milk and the strong emotional bonding between mother and child. Studies also relate breastfeeding with high-scoring achievements in cognitive tests and in motor and mental development [29, 30]. A cluster-randomized trial conducted interventions for breastfeeding promotion and found significant results for children’s long-term outcomes in health and neurological development [31].

The risk of developmental delay was found to be increased during the first 2 years of life. It has also been observed previously that, if an infant suffers from malnutrition during early childhood, the risk of developmental delay is enhanced. Furthermore, this is an indication of serious physical or psycho-social problems. Development during infancy and the toddler period is rapid and easily influenced by environmental and socio-demographic variables [1, 27, 29].

Effective community-based management of acute malnutrition, including RUTF, has gained a good reputation for treating children with SAM at the centres. Several individual-level factors (such as parental education and low socioeconomic status), facility-level factors (including distance from OTP centres, poor-quality public health infrastructure, untrained community-based healthcare workers, the palatability of RUTF), and community-level factors (such as lack of community awareness and knowledge) have proved to be potential barriers in treating children with SAM [32].

In contrast to other studies, the history of a child’s close contact with a TB smear-positive adult patient at home or in near surroundings was found to be significant in the present study. In order to understand this phenomenon, more in-depth research is required. One explanation might be that these children could have had undiagnosed latent or active TB due to malnutrition, low immune status [2, 10], or the contact with smear-positive TB patients. Therefore, a complete physical examination along with laboratory investigations is required for the diagnosis of TB in these children.

### Strengths and limitations

As well as the missing information on TB status of the children in this study, another major limitation is the cross-sectional design. Since the developmental assessment was taken at a time when acute malnutrition was already present, our data cannot determine whether the discovered deficits are reversible with therapeutic feeding or more permanent. Monitoring the children’s developmental and nutritional status over a longer time period would deliver greater insight due to the dynamic nature of growth and development. The strength of our study is that we have used the DDST-II, which is a validated scale for the developmental assessment of children. Furthermore, this assessment was conducted by well-trained medical staff using established protocols. Moreover, to our knowledge, this is the first study in rural areas of Punjab, Pakistan, to investigate the loss of developmental potential in children with SAM.

## Conclusion

Children with severe acute malnutrition have a high prevalence of suspected global developmental delay. Thus, timely identification and proper management of developmental delays in children should be reinforced in order to meet optimal growth and development needs. Developmental delay and malnutrition are two major interrelated public health problems, which hinder the achievement of the SDGs for children in low-resource countries. The health and nutritional status of these children have been adversely affected by poor feeding practices. This has directly influenced the children’s physical and mental development. The results of this study will be helpful for policymakers seeking to develop strategies for the prevention and treatment of growth failure and developmental delay in these vulnerable malnourished children.

## Data Availability

Data is available from corresponding author upon reasonable request.

## Abbreviations

AOR: Adjusted odds ratios
CI: Confidence interval
DDST-II: Denver Development Screening Tool II
MICS: Multiple Indicator Cluster Survey
MUAC: Mid-upper arm circumference
OR: Odds ratio
OTP: Outpatient therapeutic programs
PKR: Pakistani Rupees
RUTF: Ready-to-use therapeutic food
SAM: Severe acute malnutrition
SC: Stabilization centre
SDG: Sustainable Development Goal
SPSS: Statistical Package for Social Sciences
TB: Tuberculosis
WHO: World Health Organization
WHZ: Weight-for-height z-score
